# Co-Plasticization of Starch with Glycerol and Isosorbide: Effect on Retrogradation in Thermo-Plastic Cassava Starch Films

**DOI:** 10.3390/polym15092104

**Published:** 2023-04-28

**Authors:** Rudy A. Gómez-López, Camilo E. Montilla-Buitrago, Héctor S. Villada-Castillo, Aidé Sáenz-Galindo, Felipe Avalos-Belmontes, Liliana Serna-Cock

**Affiliations:** 1 Grupo de Investigación Ciencia y Tecnología de Biomoléculas de Interés Agroindustrial, (CYTBIA), Departamento de Ingeniería Agroindustrial, Facultad de Ciencias Agrarias, Universidad del Cauca, Cauca 190017, Colombia; 2 Facultad de Ciencias Químicas, Universidad Autónoma de Coahuila, Boulevard Venustiano Carranza y José Cárdenas Valdés, Colonia República, Saltillo 25280, México; 3 Facultad de Ingeniería y Administración, Universidad Nacional de Colombia Sede Palmira, Palmira 763533, Colombia

**Keywords:** thermoplastic starch, retrogradation, extrusion, co-plasticization, isosorbide

## Abstract

Thermoplastic starch (TPS) has emerged as an essential alternative to produce environmentally friendly packaging; however, retrogradation is a disadvantage that affects its shelf life. This study analyzed the co-plasticizing effect of isosorbide on the mechanical, thermal, physicochemical, and microstructural properties and the retrogradation of films obtained by blown film extrusion from thermoplasticized starch with mixtures of glycerol and isosorbide in different ratios (3:0, 2:1, 1:2, and 0:3, respectively). The results showed that the higher concentration of isosorbide significantly increased the tensile strength; however, it reduced the elongation. Retrogradation modeled using the Avrami equation showed that the presence of isosorbide reduced the retrogradation rate (k) and modified the recrystallization mechanism (n). The relative crystallinity in the plasticized TPS films was reduced to 89%, and the adsorption significantly decreased. Isosorbide was very important in reducing the retrogradation of TPS. The best performance was obtained with the 2:1 ratio of glycerol/isosorbide due to the synergistic effect between the plasticizers. The results would allow tuning the properties of TPS films by combining glycerol/isosorbide in different ratios, which enables the design of materials tailored to potential application requirements.

## 1. Introduction

There are considerable concerns about synthetic plastics, which have prompted research into new and more environmentally friendly polymers [1]. In recent years, the development of polymers from renewable sources such as starch has attracted the attention of researchers due to their origin, biodegradability, low cost, and ease of handling [2]. Starch in its native form is partially crystalline [3] and cannot be considered a thermoplastic polymer due to intermolecular and intramolecular hydrogen-bond interactions in the amylose and amylopectin chains [4,5]. Therefore, it cannot be processed using conventional technologies to produce plastic materials [6,7]. The incorporation of plasticizers helps to overcome this problem [8]. The primary function of plasticizers is to reduce the interaction between polymer chains, decreasing their intra- and intermolecular forces, which promotes the mobility, flexibility, elongation, and ductility of plasticized materials [9]. This is possible because plasticizers have polar groups in their structure (OH, COOH, and NH_2_ groups) with the ability to form hydrogen-bond-like interactions with the –OH of the glycosidic units of the polysaccharide chains [10]. The use of polyols [11], amines [9], amides [12], carboxylic acids or salts [9], ionic liquids [13], eutectic solvents [14], amino acids [15], sugars [16], and sugar-based mixtures (fructose, glucose, sucrose, and glycerol) have been reported [17]. Co-plasticization is a strategy in which the properties of two or more plasticizers are combined to produce a synergistic effect, minimizing their disadvantages and enhancing their benefits [18,19].

Glycerol is the most widely used plasticizer for obtaining thermoplastic starch due to its high boiling point, high solubility in starch chains, low cost, and availability [11]. However, its use has a disadvantage due to the high rate of retrogradation [8].

Several studies have investigated the mixing of plasticizers to transform thermoplastic starch [8,11,20,21]. The sorbitol–glycerol co-plasticization approach has helped to find a balance between the microstructural and thermo-mechanical properties of plasticized starch because the problems caused by the weak interaction of glycerol with the starch chains are reduced; thus, the addition of sorbitol provides a higher modulus of elasticity (*E*), thermal stability, and resistance to retrogradation [11]. A study has also been reported using the addition of sugars (sucrose, fructose, and glucose) as co-plasticizers with glycerol, showing that the *E* increased. At the same time, the glass transition temperature (*T_g_*) decreased, and the microstructure of the films was more homogeneous. By co-plasticizing sugar–glycerol, it is possible to reduce the cohesive forces of the polymer, demonstrating efficient plasticization [21]. In another study by Mikus et al., glycerol was mixed with sorbitol, in which there was a balance between the mobility of the chains promoted by the presence of glycerol and an increase in molecular cohesion provided by sorbitol [20]. Schmitt et al. used starch with glycerol–sorbitol mixtures by extrusion and injection that produced mechanical properties intermediate between materials plasticized with a single plasticizer [8]. In addition, it was reported that urea/ethanolamine blends led to balanced mechanical properties with less variation during storage time [8].

Isosorbide is a fused bicyclic diol and one of the three isomeric forms of 1.4–3.6 dianhydrohexitol characterized by two OH groups and two oxygen atoms intercalated on the carbon rings [22,23]. It is a promising compound as a co-plasticizer due to its biological origin, water solubility, and low melting point (approximately 65 °C). This compound is thermally stable and can withstand the process conditions required to prepare TPS [24,25]. Some studies report the use of isosorbide as a plasticizer for starch due to the interaction of starch chains and isosorbide through hydrogen bridges, suggesting an effective plasticization and a positive effect on reducing the retrogradation of the material [22,23,26,27]. The high cost of raw materials used in the production of isosorbide (corn, wheat, potato, and sorbitol) continues to be an obstacle to the growth of the co-plasticizer market [26]. Therefore, replacing a proportion of glycerol with isosorbide may be a promising alternative to improve the properties of starch-based biodegradable materials and reduce the effects of time on thermoplastic starch retrogradation. In this sense, this work aimed to evaluate the effect of co-plasticization of starch with glycerol and isosorbide on the properties of thermoplastic cassava starch films and demonstrate their ability to reduce the retrogradation process.

## 2. Materials and Methods

### 2.1. Materials

The materials used were cassava starch with 12% water on a wet basis (provided by CODIPSA, Asunción, Paraguay), commercial grade glycerol with 99.5% purity (provided by DISAN, Cali, Colombia), and isosorbide with 98.5% purity (supplied by Haihang Industry Co. Ltd., Jinan City, China).

### 2.2. Sample Preparation

The starch was dried at 80 °C for 16 h until it reached a humidity of less than 2%. The dried starch was mixed with plasticizers: glycerol and isosorbide in a ratio of 70:30 (starch/plasticizer). For co-plasticization (glycerol–isosorbide), different mixtures were made in the proportions presented in Table 1**.** The starch with the different proportions of plasticizers was mixed for 10 min in a KITCHEN AID (Benton Harbor, MI, USA) mixer and stored in hermetically sealed PET containers for 48 h [27,28,29]. The obtained starch was processed in a single screw extruder (compression ratio 5:1 and L/D of 25) (HAAKE™ PolyLab™ QC, Thermo Fisher Scientific, Herzogenaurach, Germany) and adapted to a bead die and a nozzle with a 3 mm diameter orifice. The process conditions are described in Table 1. The material obtained was granulated and stored in airtight containers until further processing.

The pellets were extruded to obtain films by extrusion blowing with a single screw extruder coupled to a blow die. The temperature profile was 130, 157, 153, and 149 °C from the feeding zone to the die. The speed was 35 rpm. The films obtained were stored in a chamber at an average ambient temperature of 20 °C and relative humidity (RH) of 60%.

### 2.3. Mechanical Properties of Tension

The tests were performed according to norm ASTM D-882-10 on a universal machine (Shimadzu EZ-L, Kyoto, Japan) at a deformation speed of 25 mm/min. The jaw spacing was 50 mm. The product obtained was cut in the machine direction (DM) and transverse to the machine (DT), with dimensions 20 mm wide and 70 mm long. The tests were carried out at room temperature.

### 2.4. Moisture Absorption

This method dried TPS samples cut to 20 mm × 50 mm in a forced convection oven at 80 °C for 24 h. The initial weight of each sample was taken, and the samples were immediately placed in a chamber with a relative humidity of 60% and 90%. Moisture contents of 60 and 90% were fixed using saturated solutions of magnesium nitrate (Mg(NO_3_)_2_) and potassium chloride (KCl), respectively. All solutions were prepared with distilled water. Samples were weighed periodically every 24 h for the first 8 days and then at 15 and 30 days of storage. The percentage of moisture absorption (*H*) expressed in percent was calculated according to Equation (1) below, where *W_t_* is the weight at time *t*, and *W_i_* is the weight in weight of dry material:

(1) $$H\;\left(\%\right)=\left(\frac{W_{t}-Wi}{W_{i}}\right)\times 100$$

### 2.5. Fourier Transform Infrared Spectroscopy (FTIR)

The films and raw materials were analyzed by Fourier transform infrared spectroscopy with a spectrometer (IRAffinity-1S, Shimadzu, Japan) coupled to an ATR device. The spectra of all samples were recorded with 4 cm^−1^ resolution in the spectral range of 500–4000 cm^−1^.

### 2.6. X-ray Diffraction (XRD)

The crystallinity of the starch and TPS films was analyzed by X-ray diffraction. An XRD diffractometer (Panalytical, Empyrean) was used at a current of 30 mA and a voltage of 40 kV. Scans were performed at 2ϴ between 7 and 40°. Relative crystallinity (*RC*) was estimated using Equation (2) below, where *C_A_* corresponds to the crystalline area, and *A_A_* is an amorphous area:

(2) $$RC\;\left(\%\right)=\left(\frac{C_{A}}{C_{A}-A_{A}}\right)\times 100$$

### 2.7. Differential Scanning Calorimetry (DSC)

Phase transitions were performed according to norm D3418-08 using a TA Instruments Q20 calorimeter (New Castle, DE, USA). A scan was performed from −60 °C to 250 °C at a heating rate of 20 °C/min. The parameters of retrogradation kinetics were calculated from the enthalpy results (∆*H*). The degree of retrogradation (*DR*) was calculated by Equation (3) [30]:

(3) $$DR=\frac{\Delta H_{t}-\Delta H_{0}}{\Delta H_{\infty }-\Delta H_{0}}$$

where ∆*H_t_* is the melting enthalpy (J/g) of the sample after retrograding for different times, ∆*H*_0_ is the melting enthalpy (J/g) of the sample without retrogradation, and ∆*H_∞_* is the melting enthalpy (J/g) of the sample after retrograding for 30 days. The analysis of the retrogradation kinetics was carried out by applying the theory of Avrami, expressed in Equations (4) and (5) [30]:

(4) $$DR=1-\exp\left(-kt^{n}\right)$$

(5) ln[−ln(1−DR)]=lnk+nlnt

where *DR* is the degree of retrogradation, and *k* and *n* indicate the crystallization rate constant and Avrami exponent, respectively. The ln *k* corresponds to the intersection, and the *n* value is the slope of plotting ln[−ln(1−DR)] versus  ln (t).

### 2.8. Thermogravimetric Analysis (TGA)

The TGA was performed by norm ASTM E1131 (2008) using a thermogravimetric analyzer TA Instruments TGA Q50 (New Castle, DE, USA). Samples of 4 to 6 mg were deposited in a platinum capsule inside the sample holder of the equipment. Heating was performed from room temperature to 600 °C at a heating rate of 20 °C/min in a nitrogen atmosphere.

### 2.9. Scanning Electron Microscopy Morphology (SEM)

The morphology of the films obtained was analyzed on the surface using a Flield-emision scanning electron microscope (FE-SEM) Hitachi UHR 8010 (Hitachi, Tokyo, Japan). The samples were vacuum dried at a pressure of 25 psi and gold–palladium coated using a Quorum machine Q150-ES (Quorum International, Fort Worth, TX, USA).

### 2.10. Statistical Analysis

The experimental results of the mechanical tension properties were analyzed by ANOVA. Tukey’s test (*p*-value < 0.05) was used to evaluate the difference between the mean values of the mechanical properties of the films.

## 3. Results and Discussion

### 3.1. Mechanical Properties of Tension

While obtaining the sample, films were obtained with all the treatments except for TPSi-30, where isosorbide was the only plasticizer, so it was impossible to analyze the samples; this was because the 70/30 starch plasticizer mixture generated an excessive increase in torque, which exceeded the maximum allowed by the equipment. This phenomenon has been reported by other authors, who attribute it to an increase in the viscosity of the molten material caused by a low concentration of plasticizer to form hydrogen bonds with the starch, which decreases the mobility of the chains [22].

**Figure 1:**
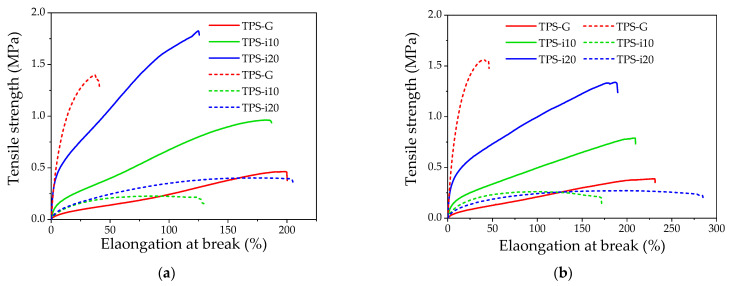
Mechanical properties of TPS films co-plasticized with glycerol/isosorbide: (**a**) orientation in the machine direction; (**b**) orientation in the transverse direction. The solid line and dashed line correspond to day 2 and day 30 of the conditioning of the samples, respectively.

The obtained results are compiled in Figure 1 and Table 2. Figure 1 shows the extruded films’ longitudinal and transverse tensile strength vs. elongation at break. The curves in Figure 1 were built with the average of the data collected for each treatment to show the tendency of the mechanical behavior of the material. Previously, the films were aged at different periods according to the experimental section. Here, it is observed that as the amount of isosorbide in the film formulation increases, the stress increases and the strain decreases. The use of isosorbide caused an increase in the elastic modulus (*E*). The E value was twice higher in the case of TPSi-10 and up to four times higher for TPS-i20 films. The same behavior was observed for the tensile strength (*σ*) values.

In contrast, the strain value (*Ɛ*) decreased by 6% for TPS-i10 and 37% for TPS-i20; this indicates that the behavior of films with isosorbide tends to be stiffer, which is in agreement with the results reported by other authors [23,27,31]. González et al. reported values for *E*, *σ*, and *Ɛ* of 55 MPa, 2.7 MPa, and 72.4%, respectively, for isosorbide-plasticized films obtained by extrusion and compression molding. The above values were higher than those observed for films plasticized with glycerol [23]. In another study by González et al., values for *E*, *σ*, and *Ɛ* of 8.7 MPa, 2.7 MPa, and 142.4%, respectively, were reported. However, in the latter case, the TPS preparation technique was by casting. In both cases, the authors attributed this behavior to the compatibility of the bonds formed between plasticizer and starch [23,27]. The differences between the values reported in the scientific literature and those found in this research could be related to factors such as the TPS preparation technique, amount of plasticizer, and plasticizing system, among other factors.

The results show that the stiffness, *E*, and strength (*σ*) (Table 2) of films plasticized only with glycerol increased significantly (*p*-value < 0.05) after 30 days of storage, while the strain (*Ɛ)* was reduced by approximately 80%; this suggests that the TPS-G materials underwent a higher degree of retrogradation, due to the reorganization of the structures that had been destroyed during starch extrusion and plasticization [32]. In addition to the molecular structure of starch, the ability of the plasticizer and polymer to form hydrogen bridges plays an important role in retrogradation [8,33]. The stronger the hydrogen bridge energy, the less recrystallization will occur during storage [34]. As previously cited, TPS-G films showed a higher retrogradation rate than samples containing some proportion of isosorbide. In this case, retrogradation is favored by the more significant amount of water, which acts as a plasticizer promoting retrogradation [11,35].

In the TPS-i10 films, after 30 days of storage, the *E* value did not undergo considerable changes. In TPS-i20, the *E* value for day 30 was reduced by 70%. Tensile strength was the parameter most affected by time, with a decrease of 76 and 77% (for TPS-i10 and TPS-i20, respectively). With the 1:2 glycerol/isosorbide ratio, the elongation of the films increased with time and reached the highest value (203.3%). In general, the TPS-i10 films (glycerol/isosorbide ratio of 2:1) were the least susceptible to time-induced changes, which could indicate a lower degree of retrogradation. Among the polyols, sorbitol has been shown to slow the rate of recrystallization best in the TPS [8] because it decreases water absorption and postpones the formation of V_H_ crystals, which contain more water molecules [11]. Isosorbide has been found to prevent crystal structure change after nine months of storage [23,31]. This agrees with XRD results, where TPS-G films had the highest increase in crystallinity. In addition, co-plasticization has been reported to be an effective strategy to prevent retrogradation, preventing the material from becoming stiff and less ductile [8].

### 3.2. Moisture Absorption

**Figure 2:**
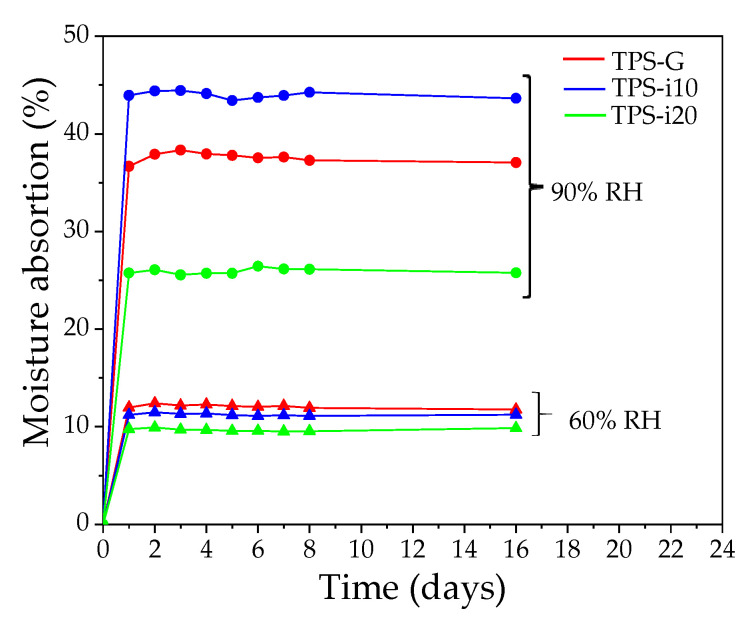
Moisture-absorption curves for TPS films at different %RH.

Figure 2 shows the behavior of TPS-G, TPS-i10, and TPS-i20 in moisture absorption versus time plots. The water-absorption capacity of TPS films co-plasticized with different glycerol/isosorbide ratios stored in environments with two relative humidities (60 and 90% RH) was evaluated. The amount of moisture absorbed varied according to the RH of the environment and the co-plasticization system. The TPS samples showed rapid absorption in the first 24 h of storage. Subsequently, the water absorption rate decreased notably, remaining relatively stable until the last day of measurement. This behavior is in agreement with other results reported by other authors [36,37]. At 60% RH, moisture absorption ranged from 9 to 12% and was much lower than at 90% RH. Although the difference in the amount of water absorbed at 60% RH was minimal, it is observed to decrease with an increasing amount of isosorbide. In the 90% RH environment, the amount of water absorbed by the films increased on average by almost three times. This behavior is attributed to the larger molecular size of the isosorbide and the lower amount of OH groups available to water, which allows for lower affinity to water [38,39].

Few studies have reported the water-absorption capacity of starch-based materials plasticized with isosorbide. It has been reported that films plasticized with isosorbide showed lower water absorption than when glycerol was used [31]. The lowest water absorption was 4.5% at 25% RH, while the highest was 22.8% at 75% RH. At 50% RH, the isosorbide-containing films absorbed 8.8% water [31]. In films obtained by casting [23], it has been found that after four weeks of storage at 43% RH, the films plasticized with 35% isosorbide went from having a wetting of 4.71 to 3.87%, while the films plasticized with glycerol went from 7.68 to 5.96% of absorbed water. This behavior was attributed to more significant interaction and homogeneity of the isosorbide-plasticized films [23].

### 3.3. Fourier Transform Infrared Spectroscopy (FTIR)

**Figure 3:**
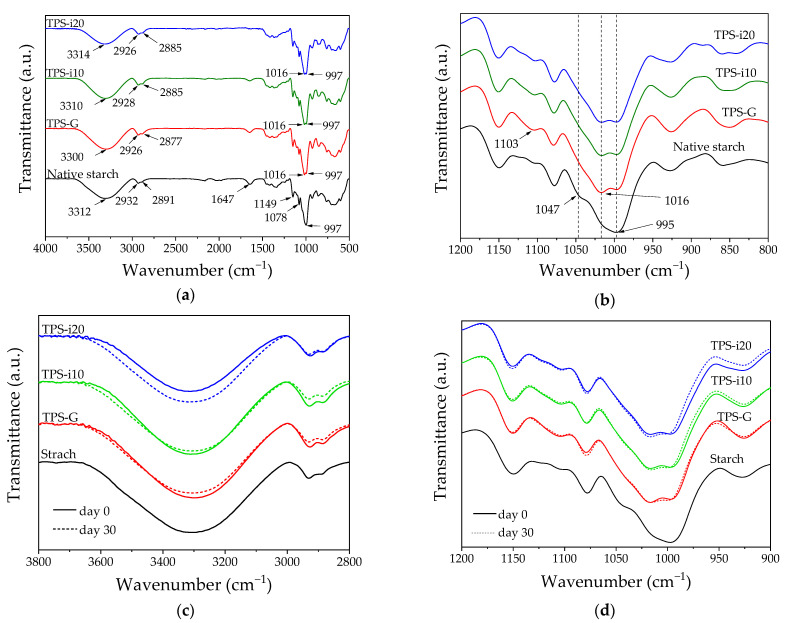
FTIR−ATR spectra of native starch and co−plasticized TPS films for day 0: (**a**) 4000–500 cm^−1^; (**b**) 1200–800 cm^−1^; and for days 0 and 30: (**c**) 3800–2800 cm^−1^; (**d**) 1200–900 cm^−1^.

One of the functions of plasticizers is to break the hydrogen-bonding forces existing in native starch to convert it into a thermoplastic material [8,40]. Infrared spectroscopy was used to estimate the influence of the type and amount of plasticizer. Figure 3 shows the FTIR-ATR spectra corresponding to native starch and the thermoplastic starch films evaluated.

Three absorption bands were observed at 3312, 1149, and 997 cm^− 1^, characteristic of starch [41]. The absorption band between 3600 and 3100 cm^−1^ corresponds to the stretching of the free OH groups of the starch structure [15]. It is associated with forming intra- and intermolecular hydrogen bonds between the starch chains [30]. This band shifted slightly in the TPS to a lower wavenumber (3300 cm^–1^) when the starch was plasticized with glycerol. Other authors have observed this behavior in potato starch materials plasticized with glycerol [42]. The addition of isosorbide increased the wavenumber assigned to stretching the O–H bond. This behavior does not agree with the results of other investigations, in which it has been reported that a higher concentration of plasticizer decreases the wave number and increases the intensity of the peak related to the hydroxyl group; this indicates a better interaction between the plasticizer and the starch chains since the number of hydrogen bridges is increased [43]; however, other authors have reported that the plasticizer composition does not significantly alter the band related to the hydroxyl group [8,17]. The absorption band at 1647 cm^−1^ in starch decreased in intensity in the TPS as the isosorbide content increased; this is related to less water absorbed by the amorphous regions of the plasticized starch [44] since this band is associated with the vibration of water bonds [30], as verified by absorption tests.

In the region from 1200 to 800 cm^−1^ (Figure 3b), changes were observed in the characteristic absorption bands of native starch (1149, 1078, and 997 cm^−1^ and two undefined shoulders at 1047 and 1016 cm^−1^), which could indicate the molecular interaction between plasticizer and starch. The bands at 1149 cm^−1^ and 1078 cm^−1^ correspond to the C–O stretching of the C–O–H group. The absorption band between 920 and 936 cm^1^ was assigned to the C–O bond stretching vibration of the C–O–C α(1,6) bond between the anhydroglucose rings of native starch [45]. In the TPS samples, the band at 1016 cm^−1^ corresponding to the C–O stretching of the C–O–C group of the starch anhydroglucose ring was intensified [11,35]; this is caused by newly formed hydrogen bonds between plasticizers and the C–O group of native starch [11,46], indicating stable hydrogen bonds between the plasticizer and both O corresponding to C–O–H and the O on the O–C of the anhydroglucose ring in starch molecules [46]. The absorption band between 1016 and 1021 cm^−1^ is associated with plasticization [21], and its appearance indicates low crystallinity [15]. A new band located at 1103 cm^–1^ was observed in the TPS, corresponding to glycerol functional groups [47]. The shoulder located around 1047 cm^−1^ decreased almost entirely when starch was plasticized, regardless of the type of plasticizer. The 1047 cm^−1^ absorption band is sensitive to the number of starch crystals [47], since the native starch crystallinity may have been destroyed and replaced by amorphous zones due to the action of the plasticizers (glycerol and isosorbide).

The FTIR spectra of the TPS were evaluated on day 30, and their typical absorption bands did not change drastically. Although the interaction associated with the wavelength range between 3800 and 2800 cm^−1^ is not directly related to plasticization [21], in samples containing only glycerol (TPS-G), the absorption band shifted from 3300 to 3307 cm^−1^, indicating an increase in recrystallization and an increase in the intermolecular strength of hydrogen bonds between starch chains [30]. The above behavior did not occur in the samples containing isosorbide (TPS-i10 and TPS-i20). The band located at 1149 cm^−1^ shifted towards a slightly higher wavenumber for all samples, whereas the absorption bands at 1078 and 1016 cm^−1^ did not change substantially with time. Although it has been reported that, during TPS aging, the height of these three bands increases [40,48,49], no significant changes in the transmittance of the corresponding absorption bands were observed, regardless of the plasticization system.

### 3.4. X-ray Diffraction (XRD)

**Figure 4:**
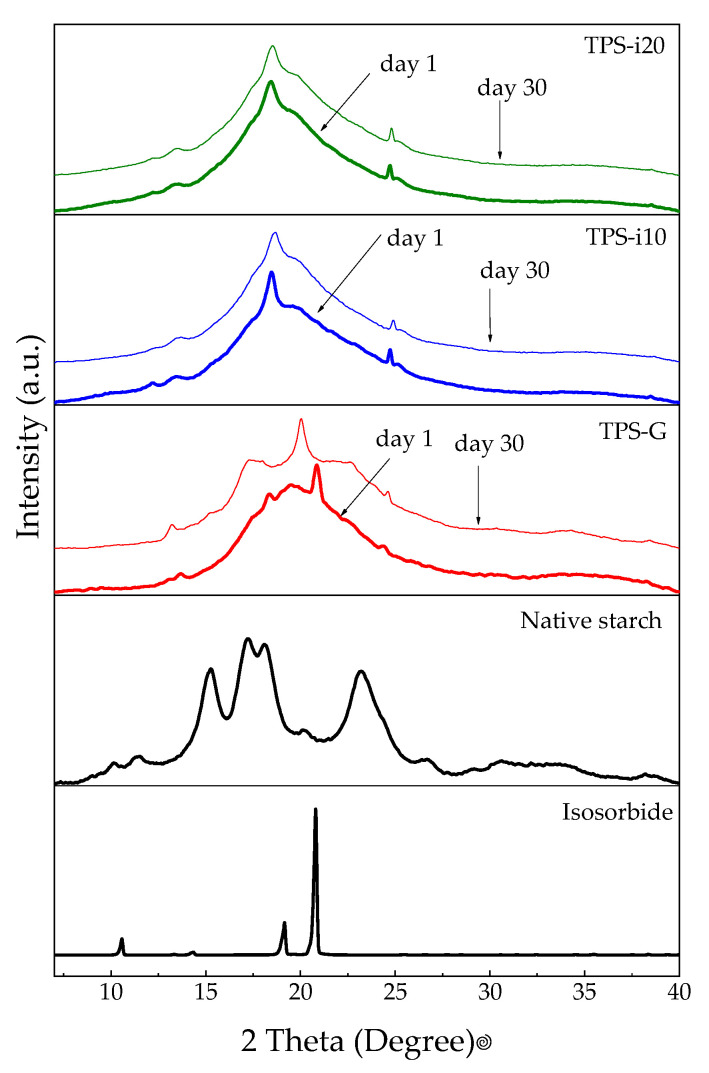
XRD diffraction spectra for TPS films and their components.

Figure 4 shows the XRD patterns of isosorbide and native starch and the different TPS films. The XRD spectrum of isosorbide was characterized by diffraction peaks mainly at 10.6, 19.1, and 20.8°, indicating the high crystallinity of this plasticizer [31]. In the spectrum of native starch, two peaks were observed at 15.3 and 23.2°. In addition, an undefined double peak at 17.3 and 18.1° corresponds to the presence of type C crystalline structures (a combination of type A and B crystals). These polymorphic structures are typical of starches from roots [50].

Compared to native starch, all samples’ amorphous area (*A_A_*) was higher. In contrast, the crystalline area (*C_A_*) decreased (Table 3), demonstrating the transformation of the semi-crystalline structure of starch into predominantly amorphous areas. Three processing-induced crystalline structures were identified: V_H_, V_A_, and E_H_ [51,52]. Differences were found between the diffraction patterns of TPS films, confirming the effect of plasticizer type and amount on TPS crystallinity. The TPS-G films present a large amorphous halo with a small peak at 18.3° due to residual crystallinity caused by incomplete plasticization of some native starch granules. The shoulder, located at 19.5°, with a smaller peak at 13.4°, corresponds to crystalline structures induced by V_H_-thermomechanical-type processing and generated by the crystallization of amylose into a simple helical structure [27,53]. In addition, a new peak was observed at 20.2° that could be attributed to V_A_-type crystalline structures, which appear immediately after processing at high temperatures and in the presence of glycerol due to the formation of hydrogen bridges between the plasticizer and the starch [30].

In the XRD diffractograms of the films co-plasticized with glycerol/isosorbide (TPS-i), a small peak at 24° was observed, attributed to residual crystallinity due to incomplete destructuring of native starch granules, which is also evident in the analysis of SEM pictures. Contrary to TPS-G films, TPS-i materials presented a single intense peak located around 18°, which is associated with E_H_-type crystallinity [31], a product of processing under higher shear stresses than those necessary to form V_H_ -type structures [27]. According to González et al., the molecular weight of isosorbide and the bulky geometry produces an increase in the viscosity of the melt, which, in turn, causes higher shear stresses during extrusion; this favors the appearance of E_H_-type structures rather than V_H_-type structures [27]. The size of the molecule could prevent more plasticizer molecules from being available for the amylose chains [22].

Table 2 shows that the relative crystallinity calculated for native starch was 29.05%. Lin et al., reported an RC value of 25.6% [54]; other authors have reported values higher than 32% [50]. However, RC values between 15 and 49% have been reported [55]. The differences between the values have been attributed to the analysis and calculation methods [55]. Films plasticized only with glycerol presented a more effective plasticization since the RC value was lower than those found with samples containing isosorbide. As the proportion of isosorbide in the TPS increased, the crystallinity increased by 19%; this indicates that, under the processing conditions employed in this investigation, the effectiveness of plasticization was reduced with increasing isosorbide. However, co-plasticization with glycerol and isosorbide could promote a synergistic effect between the plasticizers, in which the advantages of each are exploited. This synergistic effect has been reported by other authors using other plasticizers [8,11,19,21,56].

In TPS-i10 and TPS-i20 films, the type of crystalline structures (E_H_) did not change with storage time. Crystallinity (Table 3) increased by 9 and 2% in TPS-i10 and TPS-i20 films, respectively. Although the crystallinity was higher in samples with higher isosorbide content (TPS-i20), the variation with time was the lowest; this suggests that retrogradation has less of an effect when the isosorbide ratio is higher. In TPS-G, the most significant changes as a function of time were observed. The characteristic DRX peaks were modified, suggesting the appearance of other crystal structures. The diffraction patterns indicate the transformation of V_A_-type crystals into V_H_-type structures due to the retrogradation process; this is promoted by a higher amount of water absorbed in the glycerol-plasticized TPS [8]. Although the materials containing TPS-G presented a lower initial RC (Table 3), the RC increased by almost 135% after storage time; this is due to the weakening of hydrogen bonding interactions between the plasticizer and the starch and the consequent release of the starch chains by the recrystallization of amylopectin [41].

### 3.5. Differential Scanning Calorimetry (DSC)

**Figure 5:**
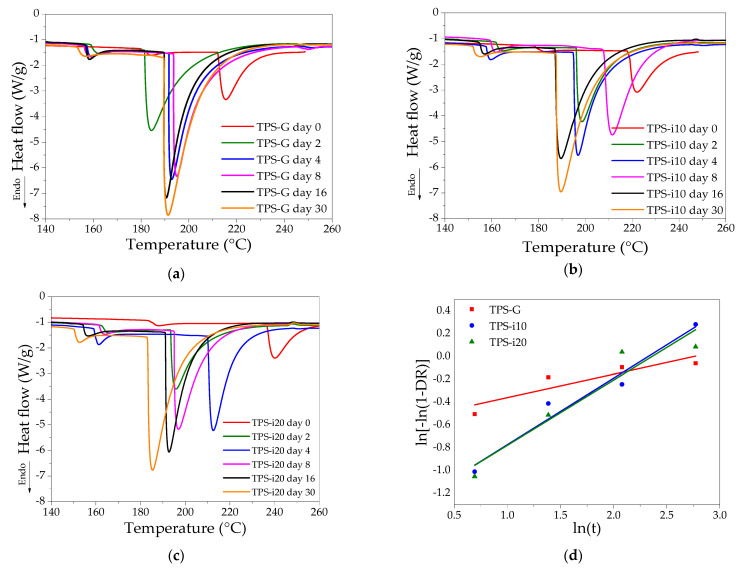
DSC thermograms and retrogradation kinetics of glycerol- and isosorbide-plasticized films: (**a**) glycerol-plasticized starch; (**b**) glycerol/isosorbide TPS-i10; (**c**) glycerol/isosorbide TPS-i20; (**d**) plots for ln[−ln(1 − DR)] versus Ln (t).

A calorimetric analysis was performed to know the thermal transitions of the TPS after processing and at different storage times. Figure 5 shows the DSC thermograms and retrogradation kinetics of the films. Endothermic melting peaks were identified, around 160 °C and above 180 °C. The first peak could be associated with the firmly bound water evaporation enthalpy, which occurs between 80 and 180 °C [57]. The second peak could be attributed to the melting the remaining crystalline domains [58] and structures resulting from the co-crystallization of amylose and amylopectin during film formation [59]. These peaks are explained by the rearrangement of the starch molecules that form crystalline structures in the presence of water and by the passage of time [30].

The partial substitution of glycerol by isosorbide drastically modified the melting temperature (*T_m_*) and the heat or melting enthalpy (∆*H_m_*), due to the interactions created by the addition of isosorbide, through hydrogen bridges between the starch and plasticizer chain [60]. Although no clear trend is observed concerning the values found for all samples, it is possible to show that the Tm of the TPS-G treatment films tends to be lower than the isosorbide-plasticized films. In addition, it is observed that the Tm of all samples decreases strongly during the first two days of storage; subsequently, it has minor variations over time until reaching a very close value for all samples on day 30. This behavior coincides with the water absorption results in Figure 2, where a substantial variation is observed in the first days and tends to stabilize with time; the effect could explain this effect that the absorbed water can have on the recrystallization of the starch chains since the reorganization of the starch chains is lower when the water content is reduced [32,61].

Moreover, the reduction of ∆*H_m_* could indicate an enhanced effect of co-plasticization produced by isosorbide incorporation, which occurs when starch–starch interactions are replaced by a new starch-plasticizer [62,63]. The molar mass and bulky geometry of isosorbide cause higher shear stress during extrusion, which promotes more significant destruction of starch granules [27]. The destruction of starch granules could allow the glycerol to penetrate the starch chains [11], increasing the plasticizing capacity of the glycerol/isosorbide system; this suggests a synergistic effect during starch plasticization, where the properties of plasticizers complement each other [11,19,21,41].

The TPS samples’ retrogradation behavior (Figure 5d) was evaluated using the Avrami model, which has been employed to analyze variations in the molecular rearrangement process [8,30,64]. The kinetic parameters of the Avrami model (Table 4) were calculated from the ∆*H_m_* values at different storage times. The coefficient of determination values (*R*^2^, 0.8173, 0.9619, and 0.9136 for TPS-G, TPS-i10, and TPS-i20, respectively) were close to 1, indicating a high fit of the experimental data in the model. However, the fit for the TPS-G treatment was lower, possibly due to the high moisture absorption capacity mainly produced in the first days of storage, which causes a rapid retrogradation [8]. The Avrami exponent (*n*) provides qualitative information about nucleation and crystallite growth [65]. The values of *n* were less than 1 in all samples, being lower for TPS-G. In TPS-i10 and TPS-i20 films, the exponent *n* was almost three times higher than TPS-G, which shows that the incorporation of isosorbide influenced the nucleation mechanism and crystallization processes during TPS retrogradation.

These observations are consistent with the XRD results, suggesting the formation of different crystalline structures according to the plasticizer. Values of *n* less than one could indicate that the formation of nucleation points followed an instantaneous mechanism during starch recrystallization, and the latter occurred mainly in the initial stage of storage [66]. The crystallization rate (*k*) decreased drastically with the addition of isosorbide, with similar values for TPS-i10 and TPS-i20 films, indicating a reduction in the retrogradation rate [64]; this is possibly due to plasticizer–starch interactions and the large size of the isosorbide molecule restricting the movement of the starch chains [31], as evidenced by FTIR spectra. In addition, this behavior could have been favored by the low amount of water adsorbed in the isosorbide-containing films. The low amount of water in the films reduces the short- and long-term rearrangement capacity of amylose and amylopectin, respectively [41,67]. Similar *n* and *k* values in order of magnitude have been reported for glycerol-plasticized and SiO_2_-added TPS films obtained by compression molding [30]. In films extruded with different plasticizers (glycerol, urea, and sorbitol), the *k* values calculated based on the storage modulus were much lower. However, the *k* for the TPS containing glycerol was higher than the other plasticizers [8], which coincides with the behavior of the results found in this study.

### 3.6. Thermogravimetric Analysis (TGA)

**Figure 6:**
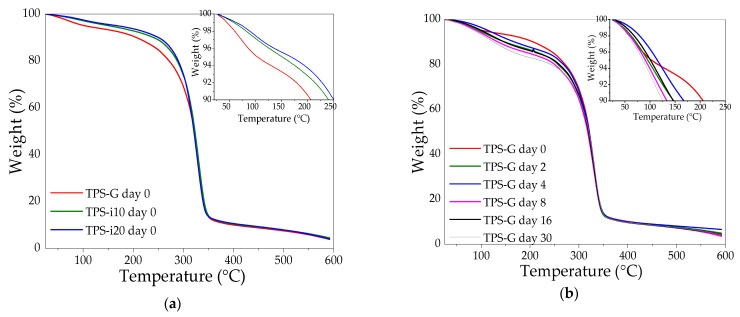

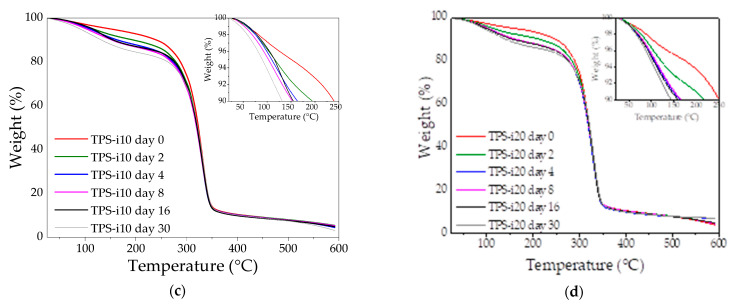
Thermogravimetric curves at day 0: (**a**) TPS films; and day 0–30: (**b**) TPS-G, (**c**) TPS-i10, (**d**) TPS-i20.

Thermogravimetric analysis was performed to study the influence of the plasticizer on the thermal degradation of TPS films co-plasticized with glycerol/isosorbide. The results were plotted using TGA curves (Figure 6). The thermal degradation behavior at day 0 (Figure 6a) was very similar for all samples analyzed. Mainly three stages were distinguished, with some variations according to the co-plasticization system. The first stage, produced around 100 °C, corresponds to water loss, although it is poorly visible due to the low humidity of the sample. The second stage corresponds to the thermal degradation of the phase with high glycerol and isosorbide content, which occurs between 190 and 320 °C, and the third stage is observed at temperatures above 320 °C, where thermal decomposition of the starch occurs [22,23,31].

The data extracted from the TGA curves are summarized in Table 5. Comparison parameters were established: T_10%_, T_50%_, and T_90%,_ indicating the temperature at which 10%, 50%, and 90% mass were lost, respectively. T_max_ is the maximum degradation temperature. The residue (%) is the fraction remaining after heating to 591.5 °C (maximum test temperature). The most significant changes were observed in the first stage of thermal degradation (T_10%_, integrated plot in Figure 6a), which increased by approximately 10 to 20 °C upon the incorporation of isosorbide. An increase in T_10%_ for films containing isosorbide could be related to the lower volatility of this plasticizer but also suggests higher thermal stability of TPS-i10 and TPS-i20 films [27].

Previous studies indicate that isosorbide effectively integrates starch and plasticizer into a single phase, where the plasticizer molecules are trapped inside the starch [22]; this is because the interaction between isosorbide and starch chains is much larger, thus making plasticization more efficient [31]. In addition, the bulky geometry of the isosorbide molecule plays an essential role in this behavior. It reduces its mobility compared to glycerol, making its release after the plasticization process more complex [23].

The thermal stability of the films as a function of time (Figure 6b–d) shows significant changes mainly at the stage of volatilization of water and the phase with high plasticizer content (integrated graphs). Although T_10%_ decreased with time, indicative of a loss of thermal stability of the materials, the T_10%_ of the films containing isosorbide remained above the T_10%_ of the films containing only glycerol as the plasticizer; this suggests that the initial starch–isosorbide interactions were more stable than the starch–glycerol interactions. Lower water absorption of TPS-i films favors this behavior, since a low amount of water reduces the ability of starch chains to reorganize [32,61].

### 3.7. Scanning Electron Microscopy Morphology (SEM)

**Figure 7:**
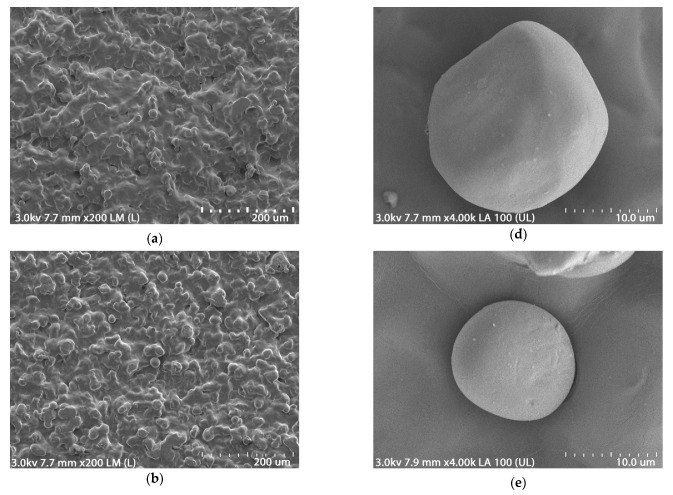

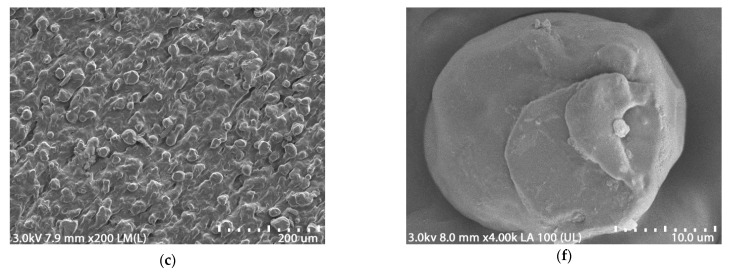
SEM micrographs of films at magnification 200×: (**a**) TPS-G; (**c**) TPS-i10; (**e**) TPS-i20; and 4000×: (**b**) TPS-G, (**d**) TPS-i10, (**f**) TPS-i20.

The surface morphology of the glycerol/isosorbide co-plasticized TPS films was studied by scanning electron microscopy (Figure 7). A compact surface was observed, with a predominantly smooth layer and variable relief product of the tension action generated during blowing and subsequent shrinkage due to retrogradation [32]. In addition, the shear stress typical of extrusion-blown processing is noted [50].

The plasticizer’s processing and presence destructured most of the starch granules. However, residual starch granule structures were observed outside the matrix (Figure 7b,d,f) and inside the matrix (Figure 7a,c,e). The residual granules result from insufficient mixing and shearing typical of single-screw extrusion [68]. This process still has room for improvement. The presence of starch granules could also indicate incomplete plasticization [69]. Starch granules that were not plasticized and remained outside the polymeric matrix were observed (Figure 7b,d,f). In them, small changes on their surface could suggest the damage caused by shearing in the extrusion and changes concerning the plasticization system employed. As the amount of isosorbide increased, the starch granules suffered more significant damage to their structure (Figure 7b,d,f); the increase in viscosity causes this and the high shear stresses when isosorbide is used [22,27,31].

## 4. Conclusions

This study obtained environmentally friendly bio-composites using raw materials of renewable origin, such as cassava starch. In addition, implementing a co-plasticization system with glycerol and isosorbide allowed complementing of the plasticizing properties of glycerol and the anti-retrogradation effect of isosorbide to obtain thermoplastic starch-based materials. Isosorbide promoted changes in the mechanical tension properties of TPS as a function of the glycerol/isosorbide ratio. Although the elongation decreased with an increasing amount of isosorbide, the presence of glycerol in the co-plasticization system allowed for obtaining a sufficiently flexible material with higher re-strength.

A partial modification of the recrystallization mechanism was identified with isosorbide, promoting the formation of predominantly E_H_-type crystalline structures, which are more stable over time and reduce the effects of retrogradation. A glycerol/isosorbide ratio of 2:1 is effective in achieving adequate starch plasticization, comparable to other plasticizing systems. This evidence suggests a synergistic effect between glycerol and isosorbide.

The results obtained are promising and helpful in the search for biodegradable materials for different applications; however, other parameters should be evaluated according to the specific application, such as oxygen or water vapor permeability, color, and transparency in the case of food packaging.

## Figures and Tables

**Table 1:** Glycerol–isosorbide ratio as starch co-plasticizers and processing conditions.

Sample	Starch (wt. %)	Plasticizer		Temperature Profile (°C)	Speed (rpm)
		Glycerol (wt. %)	Isosorbide (wt. %)		
TPS-G	70	30	0	110–155–145–109	45
TPS-i10	70	20	10		
TPS-i20	70	10	20	110–160–155–109	
TPS-i30	70	0	30		

**Table 2:** Mechanical properties of TPS films.

Sample	Day	*E* (Mpa)	*σ* (Mpa)	*Ɛ* (%)	Thickness (mm)
		Orientation in Machine Direction			
TPS-G	2	7.51 ± 1.13	0.47 ± 0.02	200 ± 7	0.31 ± 0.03
	30	123.89 ± 4.49	1.57 ± 0.05	42 ± 3	0.42 ± 0.02
TPS-i10	2	14.22 ± 1.01	0.96 ± 0.05	188 ± 3	0.24 ± 0.02
	30	14.07 ± 1.10	0.23 ± 0.02	128 ± 2	0.26 ± 0.02
TPS-i20	2	32.54 ± 3.19	1.76 ± 0.17	125 ± 21	0.21 ± 0.01
	30	9.62 ± 0.64	0.40 ± 0.01	203 ± 29	0.30 ± 0.05
		**Orientation in Transverse Direction**			
TPS-G	2	5.70 ± 1.08	0.37 ± 0.04	231 ± 47	0.31 ± 0.04
	30	140.37 ± 5.90	1.40 ± 0.07	40 ± 1	0.30 ± 0.01
TPS-i10	2	12.49 ± 0.71	0.79 ± 0.07	212 ± 15	0.25 ± 0.02
	30	12.17 ± 1.86	0.26 ± 0.01	164 ± 8	0.27 ± 0.01
TPS-i20	2	24.32 ± 0.84	1.35 ± 0.04	192 ± 14	0.22 ± 0.01
	30	7.99 ± 0.22	0.27 ± 0.01	285 ± 6	0.34 ± 0.01

**Table 3:** Amorphous area (*A_A_*), crystalline area (*C_A_*), and relative crystallinity (RC) of TPS films before and after storage time.

Sample	Time (Days)	*A_A_*	*C_A_*	RC (%)
Starch	-	5.82	2.38	29.05
TPS-G	1	7.57	0.23	2.93
	30	7.81	0.58	6.89
TPS-i10	1	7.48	0.24	3.10
	30	7.34	0.26	3.38
TPS-i20	1	7.25	0.28	3.71
	30	7.32	0.29	3.82

**Table 4:** Thermal properties and retrogradation kinetic parameters for starch-, glycerol-, and isosorbide-based films.

Sample	Parameter	Time (Day)						*n*	*k*
		0	2	4	8	16	30		
TPS-G	*T_m_* (°C)	215	184	193	195	191	191	0.21	0.52
	∆*H_m_* (J/g)	55	131	150	155	157	222		
	DR	-	0.45	0.57	0.60	0.61	-		
TPS-i10	*T_m_* (°C)	222	198	197	211	180	190	0.62	0.24
	∆*H_m_* (J/g)	52	96	123	131	159	198		
	DR	-	0.31	0.48	0.54	0.73	-		
TPS-i20	*T_m_* (°C)	240	196	213	196	193	185	0.55	0.24
	∆*H_m_* (J/g)	41	79	100	125	127	171		
	DR	-	0.29	0.45	0.65	0.66	-		

**Table 5:** TGA data for starch-, glycerol- and isosorbide-based TPS films.

Variable	Sample	Time (Days)					
		0	2	4	8	16	30
T_10%_ (°C)	TPS-G	230	148	168	134	147	125
	TPS-i10	240	194	164	153	155	132
	TPS-i20	250	217	162	166	159	143
T_50%_ (°C)	TPS-G	322	319	321	318	319	320
	TPS-i10	323	321	320	319	321	322
	TPS-i20	321	321	319	320	320	322
T_90%_ (°C)	TPS-G	411	390	402	392	388	388
	TPS-i10	412	407	404	402	394	401
	TPS-i20	421	418	395	416	406	399
T_max_ (°C)	TPS-G	331	331	331	330	331	332
	TPS-i10	331	330	330	330	330	333
	TPS-i20	328	329	328	330	329	331
Residue	TPS-G	3.6	4.9	6.5	3.5	4.3	4.2
	TPS-i10	4.5	5.4	4.4	5.2	4.8	3.0
	TPS-i20	4.0	4.6	7.1	4.5	4.8	7.1

## Data Availability

The data presented in this study are available on request from the corresponding author.
